# Malaria mortality characterization and the relationship between malaria mortality and climate in Chimoio, Mozambique

**DOI:** 10.1186/s12936-017-1866-0

**Published:** 2017-05-22

**Authors:** João Luís Ferrão, Jorge M. Mendes, Marco Painho, Sara Zacarias

**Affiliations:** 1 Faculdade de Engenharia, Universidade Católica de Moçambique, Chimoio, Mozambique; 2 NOVA Information Management School, NOVA University of Lisbon, Lisbon, Portugal; 3 Direccao Provincial de Saúde de Manica, Lisbon, Mozambique

**Keywords:** Malaria mortality, Seasonality, Spatiality, Chimoio, Precision Public Health

## Abstract

**Background:**

The United Nation’s sustainable development goal for 2030 is to eradicate the global malaria epidemic, primarily as the disease continues to be one of the major concerns for public health in sub-Saharan Africa. In 2015, the region accounted for 90% of malaria deaths. Mozambique recorded a malaria mortality rate of 42.75 (per 100,000). In Chimoio, Mozambique’s fifth largest city, malaria is the fourth leading cause of death (9.4%). Few data on malaria mortality exists in Mozambique, particularly in relation to Chimoio. The objective of this study was to characterize malaria mortality trends and its spatial distribution in Chimoio.

**Methods:**

Malaria mortality data and climate data were extracted from the Chimoio Civil Registration records, and the Regional Weather station, from 2010 to 2014. The malaria crude mortality rate was calculated. ANOVA, Tukey’s, Chi square, and time series were carried out and an intervention analysis ARIMA model developed.

**Results:**

A total of 944 malaria death cases were registered in Chimoio, 729 of these among Chimoio residents (77%). The average malaria mortality by gender was 44.9% for females and 55.1% for males. The age of death varied from 0 to 96 years, with an average age of 25.9 (SE = 0.79) years old. January presented the highest average of malaria deaths, and urban areas presented a lower crude malaria mortality rate. Rural neighbourhoods with good accessibility present the highest malaria crude mortality rate, over 85 per 100,000. Seasonal ARMA (2,0)(1,0)_12_ fitted the data although it was not able to capture malaria mortality peaks occurring during malaria outbreaks. Intervention effect properly fit the mortality peaks and reduced ARMA’s root mean square error by almost 25%.

**Conclusion:**

Malaria mortality is increasing in Chimoio; children between 1 and 4 years old represent 13% of Chimoio population, but account for 25% of malaria mortality. Malaria mortality shows seasonal and spatial characteristics. More studies should be carried out for malaria eradication in the municipality.

**Electronic supplementary material:**

The online version of this article (doi:10.1186/s12936-017-1866-0) contains supplementary material, which is available to authorized users.

## Background

One of the United Nation’s sustainable development goals for 2030 is to end the epidemics of AIDS, tuberculosis, malaria and other neglected tropical diseases [1]. Malaria continues to be one of the major concerns for public health in Africa. Sub-Saharan Africa presents a disproportionately high portion of the global malaria burden. In 2015, the region was home to 88% of malaria cases and 90% of malaria deaths. The number of malaria deaths globally was 438,000 in 2015 (Range 236,000–635,000) [2]. According to the Mozambican Ministry of Health, the country recorded over six million cases of malaria in 2015 [3] and deaths due to malaria (per 100,000) was 42.75 in 2013 [4]. Malaria killed 3245 people and is the second cause of death in the country, at 19.2% [5].

Chimoio is a municipality in the central region of Mozambique where the incidence of malaria is 20.1%, and the attributable factor 16% [6]. Malaria is the fourth leading cause of deaths in Chimoio at 9.4% [7]. Climatic factors such as temperature, relative humidity, precipitation and evaporation influences the lifecycle and development of both the mosquito vector and the parasite [8].

Understanding the trends and variation of deaths is of paramount importance for precision public health. Precision public health is a relatively new concept and its ultimate goal is to develop and implement health interventions that can benefit the right population at the right time [9]. Civil registration constitutes the most timely and accurate source of information on mortality and causes of death. In Mozambique registration of deaths falls under the Ministry of Justice [10].

Describing trends and characteristics of malaria mortality, and its relationship with climate factors, can assist in monitoring and planning resource needs of the health system and municipal management.

Geographic information systems can help to describe variations in malaria mortality and this is important to identify areas at high risk, to assist in designing appropriate interventions, or lead to further investigations to identify important risk factors [11].

As stated elsewhere, the best ways to help the living is by counting the dead. Few data on malaria mortality, trends and characteristics of malaria death exist in Mozambique, particularly in Chimoio. The few existent data are from hospitals and do not represent the entire community.

The objective of this study is to determine malaria mortality trends, characterize malaria mortality, describe its spatial distribution and variation in Chimoio, and verify its relationship with climate parameters to help local authorities in programmatic malaria activities for the prevention and eradication of the disease.

## Methods

### Study area and population

**Fig. 1:**
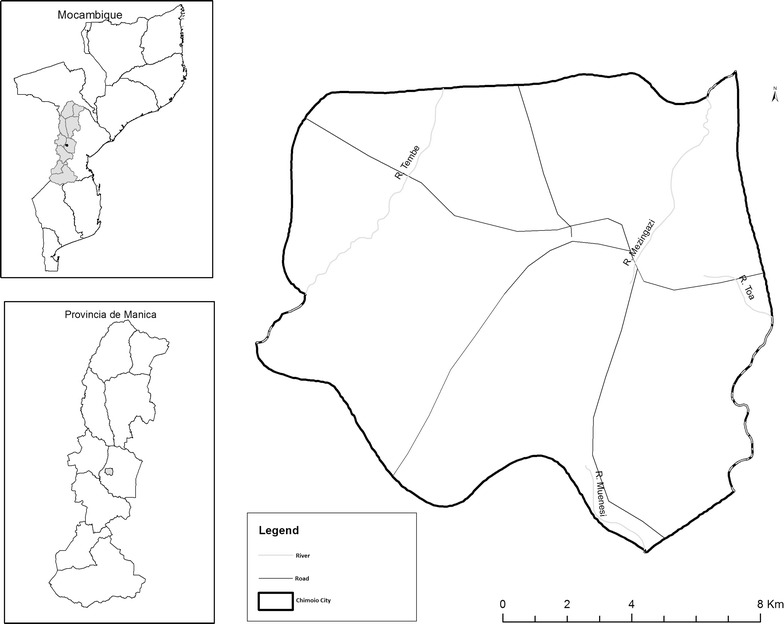
Map of Chimoio

Chimoio is a municipality of Manica Province in the centre of Mozambique, located at −19° 6′ 59 S, 33° 28′ 59 E. The current population projection by the “Instituto Nacional de Estatística” (National Statistics Institute) is 324,816, being 50.4% males and 49.6% females. The population percentage by category is: age 0 (3%), 1–4 (13%), 5–14 (28%), 15–44 (48%), 45–59 (6%) and over sixty (2%) [12]. Chimoio is divided into 33 residential areas known as “Bairros” or neighbourhoods with an area of 174 Km^2^ (Fig. 1).

### Study subjects

Death cases and monthly malaria mortality data were extracted from the Chimoio civil registration books from 2010 to 2014. Data entered in the books come from death certificates produced by qualified health personnel. The variables extracted were sex, month of death, cause of death, age, place of death, time of death, and the origin of the deceased. Population data were extracted from the population projection data by the “Instituto Nacional de Estatística” of Mozambique [13]. For malaria cases, data reported elsewhere were used [6]. Monthly climate data from 2010 to 2014 were obtained from the Chimoio Regional Weather Station and comprised of the following parameters: mean, maximum and minimum temperature (^o^C), relative humidity (%), precipitation (millimetres) and evaporation (millimetres). The evaporation data had three missing data which were imputed using nearest data as donors.

### Data analysis

The malaria crude mortality rate was calculated per malaria year by age-specific, gender and residential area (Bairro). Malaria crude mortality rate (MCMR) was calculated dividing the number of deaths-per year of residents by the total population for the same geographic area and multiplied by 100,000:

$${\text{MCMR}} = \,\frac{{{\text{Number of deaths}} {\text{-}} {\text{per year }}}}{{ {\text{Total population for the same geographic }}}} \times {100,000}$$

Age-specific malaria mortality rate was calculated dividing the number of deaths-per age per year of residents by the total age population and multiplied by 100,000 [14]. The ages (categories) used were: 0 (infants), 1–4 (Children), 5–14 (adolescents), 15–44 (young adults), 45–59 adults and over sixty (elderly).

Chi square for a proportion of gender and age-specific category was performed and Phi, Cramer’s V test was used for statistical significance. Analysis of Variance (ANOVA) was used to test difference between years and months using the following model:

1 $$Y_{ij} = \mu + t_{i} + e_{ij}$$

Intervention analysis with the specification $z_{t} = \frac{{\delta_{0} }}{1 - wB}P_{t}$, where $\left| w \right| < 1$, $B$ stands for the traditional time series backshift operator, $Bz_{t} = z_{t - 1}$, and $P_{t}$ denotes a pulse function such that $P_{t} = 0, \;t < t_{0} \;{\text{or}}\;,\; t > t_{0}$ and $P_{t} = 1, \;t = t_{0}$, where $t_{0}$ is the moment of intervention [15] was used.

All tests were performed using R 3.3.2, SPSS, IBM version 20 and Biosat 5.0. Spatial maps for year variation were produced using ArcGIS version 10.1.

## Results

### Deaths, malaria cases and malaria mortality trends in Chimoio

**Fig. 2:**
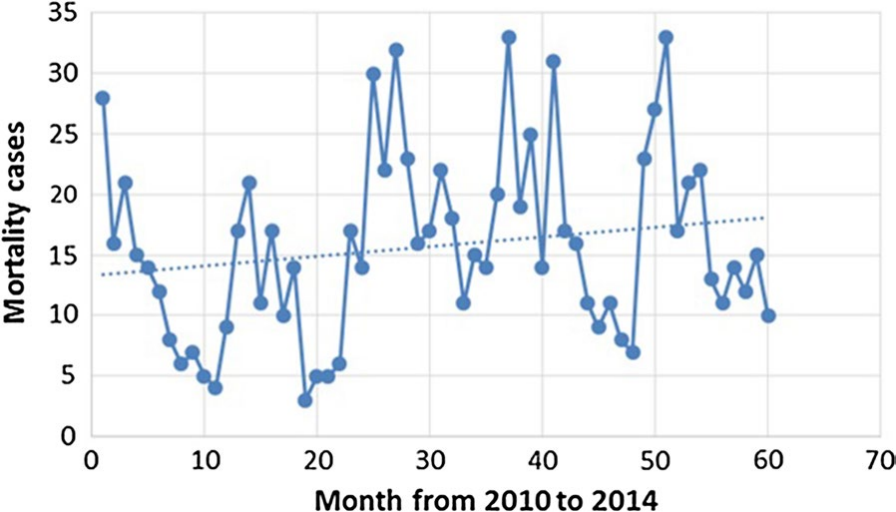
Monthly mortality trend in Chimoio between 2010 and 2014

During the period, 18,508 cases of all death causes occurred, yearly average of 3702 (SD = 137). Malaria cases were 286,583. A total of 944 malaria death cases were registered, 729 of them among Chimoio residents (77.2%). A time series plot indicates that malaria is increasing annually (Fig. 2). Year 2014 recorded the highest number of deaths 159, and the average death cases per year was 146 (SD = 38). From 2010 to 2014, the average malaria crude mortality rate (MCMR) was 51 per 100,000 (Table 1).

**Table 1:** Malaria crude mortality rate from 2010 to 2014 in Chimoio

Year	2010	2011	2012	2013	2014	Average	SD
Population	267,456	276,468	285,716	295,189	304,873	285,940	14,794
Malaria cases	41,925	47,107	52,463	60,381	84,707	57,317	16,765
All cause death	3676	3893	3509	3706	3724	3702	137
Malaria death	111	111	201	147	159	146	38
Mortality rate (per 100,000)	1374	1408	1228	1255	1221	1298	78.1
Incidence (%)	15.7	17	18.4	20.5	27.8	20	5
MCMR	41.5	40.1	70.3	49.7	52.1	51.0	12.1

*MCMR* Malaria crude mortality rate, *SD* Standard deviation

### Malaria mortality by gender and place of death

The average mortality in malaria by sex was 44.9% for females and 55.1% for males. There is no difference, (*χ* ^*2*^  =  0.415, *df*  =  1, *P*  =  0.615), between malaria mortality in females and males in Chimoio. The deaths from malaria registered in Chimoio indicated that 77% of deaths occurred at public hospital, 22% at residence and 1% at private clinics.

### Malaria death by age and age-specific

**Fig. 3:**
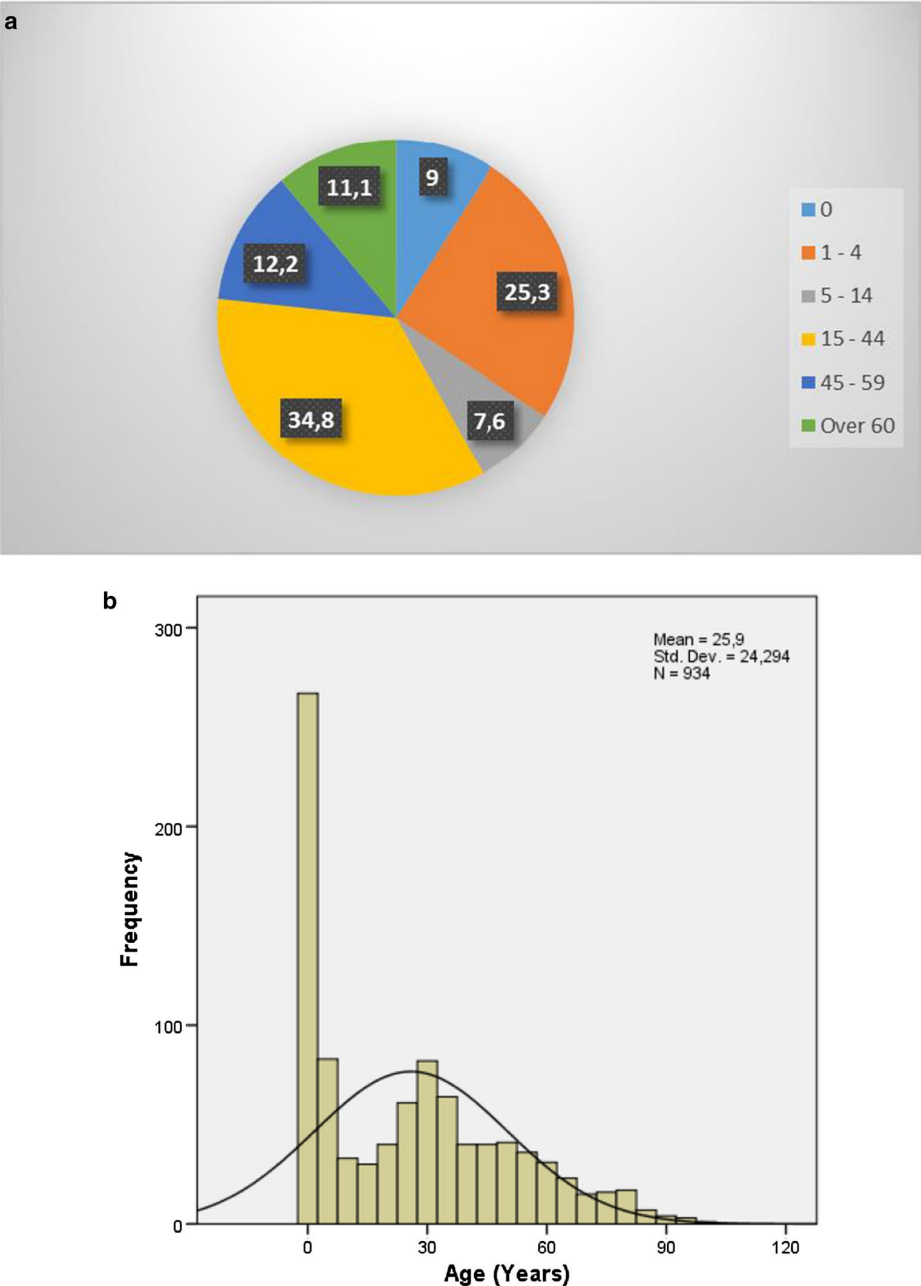
Malaria death by age. **a** Present age of death and **b** age-specific death

Figure 3 presents the malaria death by age. Figure 3a presents age of death and Fig. 3b, malaria age-specific death. The range of age of death was from 0 to 96 years, the average age of death was 25.9 years old (SE = 0.79). The first quartile (25%) of malaria deaths occurs at age of 2 and the third quartile of malaria deaths at age of 43. Out of all Chimoio residents’ registered cases, 9% were of age, 0, 25.3% were of 1–4 years old, 7.6% were of 5–14 years old, 34.8% were of 15–44 old, 12.2% were of 45–59 years old and 11.1% for the elderly. There is a difference (*χ* ^*2*^  =  15.65, *df*  =  1, *P*  <  0.001) between age categories in malaria mortality in Chimoio.

### Malaria death per year and month

**Fig. 4:**
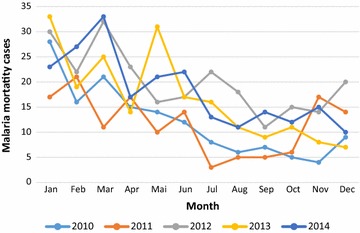
Malaria mortality trend in Chimoio per month and year

Figure 4 presents mortality trend in Chimoio per month and year. Year 2012 presented the highest number of malaria deaths, 210 (SD = 6.3) and year 2011 the lowest number of cases, 140 (SD = 11). There is a difference (*F*_(4, 59)_ = 7.91, *P* = 0.0001) in malaria mortality in Chimoio, between years. January presented the highest malaria average death, 26 (SD = 6.3), while August presented the lowest average cases of death 9 (SD = 3.5). There is a difference (*F*_(11, 59)_ = 8.12, *P* = 0.0001) in malaria mortality between months.

### Malaria mortality per time of death

**Fig. 5:**
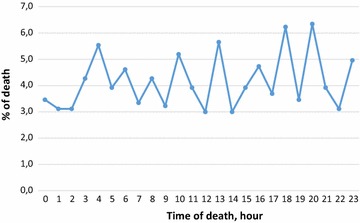
Malaria mortality per daily hour in Chimoio

Figure 5 presents the malaria mortality per hour. The highest proportion of malaria mortality was recorded in the evening, at 8:00 p.m. with 6.3% of the cases, and the lowest time of death was recorded during the day at 12:00 and 2:00 p.m. with 3% respectively. There is no difference (P > 0.05) between hours of death by malaria in Chimoio (*G* = 3.6754, *df* = 23, *P* = 0.001).

### Geographic malaria mortality variation in Chimoio

**Fig. 6:**
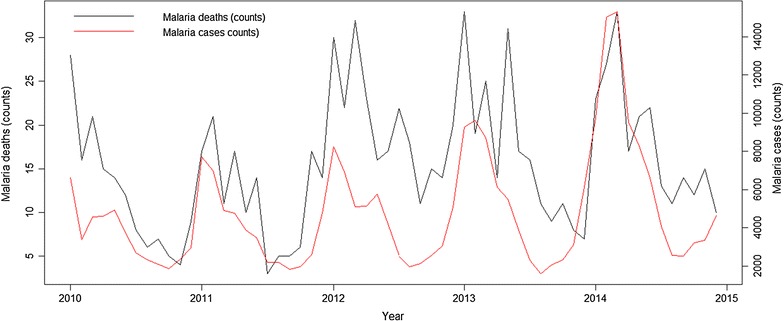
Crude malaria mortality rate per residential area

Figure 6 presents the crude malaria mortality rate per residential area. Out of 33 neighbourhoods, six bairros (18%) presented low CMMR, eleven (33%) presented moderate CMMR, ten (30%) high and six (18%) very high CMMR per 100,000. The urban neighbourhoods (low population density, Bairros 1, 2, 3) and rural neighbourhoods, with lack of accessibility (Hombwa, Chissui, Circulo Mudzigandzi, and Chianga) presented a lower malaria crude mortality rate (0–27) per 100,000. Most the neighbourhoods present a moderate malaria crude mortality rate, 27–55 per 100,000 and, rural neighbourhoods with good accessibility present the highest malaria crude mortality rate, over 85 per 100,000.

### The relationship between death by malaria mortality and climate

**Fig. 7:**
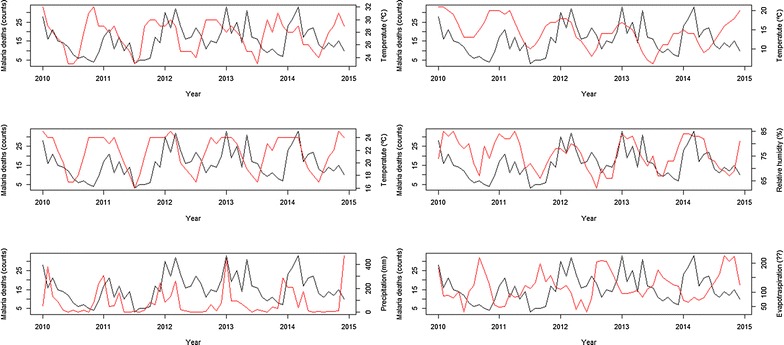
Times series of malaria deaths (*solid black line*, left y axis) and malaria cases (*solid red line*, right y axis) between 2010 and 2014

Figure 7 shows monthly malaria deaths (left y axis) and malaria cases counts (right y axis) between 2010 and 2014. As expected, deaths peak between January and March, the period of malaria outbreaks. Previous work has that shown climate factors, such as temperature, precipitation and relative humidity, are determinant to malaria outbreaks, and consequently to the number of deaths caused by malaria. Indeed malaria transmission occurs throughout the year with peaks between January and March.

**Fig. 8:**
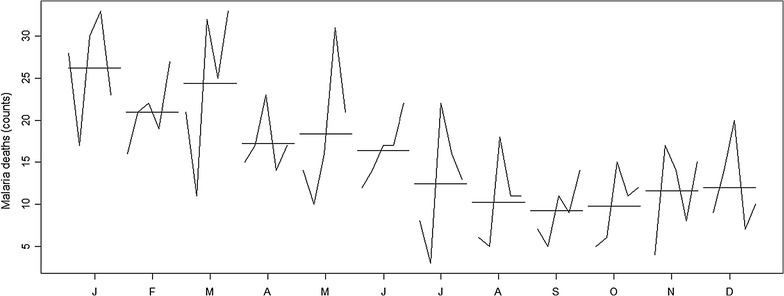
Times series of malaria deaths (*solid black line*, left y axis) and maximum, minimum and mean temperatures, relative humidity, precipitation and evapotranspiration (*solid red line*, right y axis) between 2010 and 2014

**Fig. 9:**
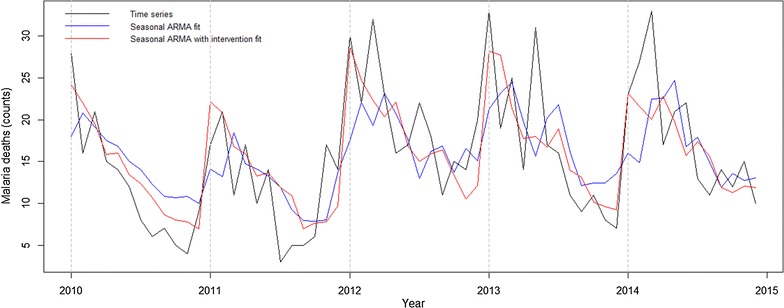
Month plot of malaria deaths. *Solid broken line* represents malaria deaths level and *solid horizontal lines* represent monthly means

Figure 8 exhibits the temporal relationship between malaria death counts and those climatic factors. Temporal behaviour of deaths and its close relationship with climatic factors suggests the extraordinary changes in the location might be properly modelled by intervention analysis (as described in [15]). Indeed, Fig. 9 shows the level of malaria deaths reaches a peak every January. The level of deaths decays then to previous levels at a decreasing rate. Following Box and Tiao [16], the specification $z_{t} = \frac{{\delta_{0} }}{1 - wB}P_{t}$ where $\left| w \right| < 1$, $B$ stands for the traditional time series backshift operator, $Bz_{t} = z_{t - 1}$, and $P_{t}$ denotes a pulse function such that $P_{t} = 0,\; t < t_{0} \;{\text{or}},\; t > t_{0}$ and $P_{t} = 1, \;t = t_{0}$, where $t_{0}$ is the moment of intervention (in this case the abrupt increase of malaria cases during malaria outbreaks every January illustrates an intervention with an abrupt temporary effect $\delta_{0}$ that gradually decays at rate $w$ with a return back to original or pre-intervention level.

A seasonal ARMA model, ARMA(2,0)(1,0)_12_ fits these data, but it is not able to capture the sudden change occurring during malaria outbreaks, despite the three statistically significant parameters. Introducing the intervention effect described above where $P_{t} = 1, \;t = {\text{January}},\; P_{t} = 1, \;{\text{otherwise}}$ allows for an improvement in the fit of death peaks. In particular, the seasonal ARMA model with intervention reduces root mean square error by almost 25% (see Additional files 1, 2).

## Discussion

The civil registration covers all registered malaria mortality cases from hospitals and from private residences. In this study, 78% of malaria deaths occurred in hospitals and the 22% at private residences. A previous study in Chimoio reported that in all-cause deaths, 86.1% of the deaths took place in hospitals and 11.7% at private residences [7]. Malaria deaths at private residences is two times greater than in all-cause of death in Chimoio. These disparities can probably be because malaria patients delay the treatment of the disease resulting in fatalities.

Trend analysis indicates that in Chimoio, cases of deaths, and malaria deaths are increasing over the years, contrary to reports in Kwazulu Natal [18], Malawi [11], and Tanzania [19] that reported decreasing cases in malaria mortality. The malaria crude mortality rate per 100,000 was 51 per 100,000, higher than the national Mozambique figure of 42.75 for 2014 [3]. In terms of malaria mortality by gender, there was no difference between malaria deaths in females and males. Similar results were reported previously by [17]. The results disagree with the findings in Kwazulu Natal and Sudan that reported higher mortality from malaria in males than in females [18, 20]. There is evidence that suggests that given equal exposure, adult men and women are equally vulnerable to malaria except for pregnant women [20]. In this study, 25% of malaria deaths occur at the age of 2, and 75% of malaria deaths at the age 43. The results are in concordance with a report on all causes of death carried out in Chimoio [7].

Age category 0 comprises 3% of the Chimoio population and recorded 9% of malaria deaths while, age category 1–4 comprises 13% of the Chimoio population, and recorded 25% of malaria deaths. This can be due to the lack of immunity in the first years of life. Similar results were reported in another seven African countries and Bangladesh [11, 21–25]. From the age of 45 onwards the proportion of deaths by malaria and, all-cause mortality is almost the same.

Malaria mortally was significantly different between month and years. Similar results of seasonality were reported in Ethiopia and Burkina Faso [24, 25] and were related to climatic conditions. January, February and March presented the highest percentage of mortality from malaria decreasing thereafter. This peak period occurs 2 months after the rainy season onset.

There was no difference in times of death from malaria in Chimoio, and this result clearly contradicts a previous report on all-cause mortality in Chimoio, that indicates that peak mortality occurs between 3:00 and 4:00 a.m. [7]. This result suggests that malaria deaths can occur at any time contrary, to other deaths that were found to peak from 3:00 to 4:00 a.m. in Chimoio.

The centre of town (low density) presents a low malaria crude mortality rate, 0–27 per 100,000 and the rural “Bairros” a very high crude mortality rate, over 80 per 100,000. This can be due to the fact that the centre has better health facilities and infrastructures which means the residents are better-off than in rural areas. Some rural neighbourhoods present low malaria mortality rates. This can be attributed to the fact those areas have poor accessibility and the residents carry out their burials without Civil Registration.

The onset of rain occurs in mid-November. This indicates that malaria occurrence has a strong association with rainfall 6–8 weeks before, coinciding, with the malaria cycle’s three components: (i) the growth of the *Anopheles* female mosquito from egg to adult to parasite transmission; (ii) the development of the *Plasmodium* parasites (gametocyte to sporozoites) that are able to infect humans; and (iii) the incubation period in the human host from infection to malaria symptoms [22]. Thus, malaria occurrence peaks can be expected 45–60 days after the onset of rain. Similar results were also found in Mozambique [4] and South Africa [18]. Increased precipitation can provide more breeding sites for mosquitoes, however excess rain can also destroy breeding sites [26].

ARMA (2,0)(1,0)_12_ fitted the data well although it was not able to capture the sudden change occurring during malaria outbreaks. Introducing the intervention effect allowed for a better fit of death peaks and the seasonal ARMA model with intervention reduced root mean square error by almost 25%. Other studies reported ARIMA (2,1,1) in Zambia, ARIMA (1,0,0) in Burundi [27], and India [14] with comparable results.

Besides the possibility that the malaria mortality was under-reported, especially in the rural areas, another limitation of this study is that it did not take into consideration malaria intervention factors such as bed net distribution and improvement of health coverage. Despite the limitations, one great strength of the study is that this is the first specific study in malaria mortality using civil registration data in Chimoio. More data from other additional data from other parts of the country are needed to generalize the results to the national level.

## Conclusion

Malaria mortality is increasing in Chimoio and strong and appropriate actions are needed to counteract the malaria deaths scenario in Chimoio. There is no difference in the malaria mortality rate between males and females. Children between 1 and 4 years old are 13% of Chimoio population, but represent 25% of malaria mortalities. The last 3 months of the rainy season (January, February and March) present more malaria mortality cases than the dry season. Urban “Bairros” in the centre of town have lower malaria crude mortality rate than the rural “Bairros”. More studies should be carried out for malaria eradication in the municipality.

## Additional files

**Additional file 1.** Anova: two-factor without replication.

**Additional file 2.** Malaria 2010.
